# Supplementary material for: Routine immunization intensification, vaccination campaigns, and measles transmission in Southern Nigeria
Source: PLoS One. 2026 Feb 18;21(2):e0341016. doi: 10.1371/journal.pone.0341016 (PMC12915958; doi:10.1371/journal.pone.0341016)
Supplement: Attachment — Submitted filename: RebutallLineByLine.pdf [file pone.0341016.s001.pdf]

Reviewer 1: This is a well-executed and insightful study that makes a clear contribution to understanding measles transmission dynamics and vaccination impact in Southern Nigeria. The modeling framework is rigorous, the analysis is transparent, and the conclusions are well supported by the data. The comparison between large-scale campaigns and the targeted 2019 intensification provides valuable policy-relevant insight into optimizing immunization strategies. The manuscript is well written, logically structured, and requires no revisions. I recommend acceptance as is.

Response: We want to thank Reviewer 1 for their time and attention, and we appreciate the positive feedback.

Reviewer 2: The authors investigate the impact of an intensified, targeted measles vaccination campaign that complemented existing supplemental immunization activities (SIAs) in reducing the susceptible population in Nigeria. Their objective is to evaluate whether these targeted campaign more effectively reach unvaccinated individuals who are typically missed by routine or prior SIA efforts.

To achieve this, the study integrates multiple data sources, including serosurveys, Multiple Indicator Cluster Surveys (MICS), Demographic and Health Surveys (DHS), and case reporting estimates, to parameterize a stochastic time-series susceptible–infected–recovered (TSIR) model. The model extends the classical TSIR framework originally introduced by Finkenstädt and Grenfell (2000) by incorporating time-varying reporting rates and demographic renewal through birth data, while drawing from nationally representative surveys, including the Nigeria Demographic and Health Surveys and Multiple Indicator Cluster Surveys to inform population and vaccination trends. This integration of demographic data and mechanistic modelling provides a data-driven reconstruction of susceptible dynamics, offering insights into how health-system improvements, campaign rollouts, and surveillance evolution may have shaped observed measles patterns. The same modelling framework was prior applied to measles dynamics in Pakistan and Somalia by the authors, where it successfully demonstrated the model's ability to disentangle true transmission changes from variations in reporting.

In the present work, the authors adapt this framework to Nigeria to quantify the effectiveness of an intensified measles campaign, estimating the efficacy per vaccine dose. Despite delivering roughly half the total number of doses compared with the standard SIA, the intensified campaign achieved a higher probability of reaching previously missed susceptible children.

This finding offers valuable insight into measles control and elimination efforts in high-burden settings, particularly those where young children transition from maternal antibody protection to susceptibility before being reached by routine or catch-up immunization. The results carry clear policy relevance for optimizing campaign design and targeting in the final stages of measles eradication as well as insights in outbreaks forecasting.

> It would be interesting to see the estimated averted case burden (similar to the authors' Somalia paper). A broader audience, including policy makers, would benefit from seeing the trajectory of cases without the intervention compared with those averted by the campaign.

Response: We want to thank Reviewer 2 for a clearly thorough reading and the context in terms of the related literature. On this specific suggestion, it is possible to make a comparable figure to Fig. 4 in the Somalia paper (Thakkar et al., *Vaccines* 2024). However, the key theme in this paper is the relative comparison of the different types of interventions, specifically the conventional campaigns relative to the 2019 IRI. We feel it would distract from that purpose to add a new counterfactual figure.
That said, the main takeaway from a Somalia-like counterfactual exploration is actually apparent in the forecast test in Fig. 3, where a delayed 2022 campaign led to an outbreak in Lagos. We've added a sentence to the caption to clarify that if the 2019 IRI was removed, we would have seen a comparable outbreak earlier.

> **Reviewer 2 continued:** The following refinements would improve readability and accessibility for a broad epidemiologic audience:
>
> Minor Comments
>
> 1. Model Construction: 1.1. Move the core model description and assumptions into the main Methods section rather than leaving them solely in the appendix. 1.2. Add a visual summary showing data inputs (births, MICS/DHS, campaigns) and model compartments and flows. This would significantly improve clarity for non-modelling readers. 1.3. Parameter table in the appendix: Include a concise parameter summary table listing each symbol, its description, unit, and epidemiologic interpretation. This addition would enhance accessibility and reproducibility.

Response: We appreciate these suggestions on clarity. Having the mathematical description of the model in the appendix, leaving the main text equation-free, was intentional. In seeking feedback from a variety of readers, including policy-focused peers and quantitative scientists, we found that this set up facilitated a range of different readings. We think this arrangement is mainly a matter of preference, and we choose to leave it as is.
On suggestions 1.2 and 1.3, the reference table of parameters is a nice idea, and we've added it to the appendix. As far as a figure, while we know that compartmental flow charts are common in the literature, in this case with only 2 compartments, it's not clear how much it would add. Moreover, in the context of stochastic models, we have found that flow charts reduce distinct processes (log-normal noise, binomial samples, Gaussian processes) ambiguously and sometimes inaccurately. Our preference is either a text description or the equations, and we don't feel like a flow chart is needed.

> **Reviewer 2, minor comments continued:** 2. Figures and visual aids: 2.1. Label all multi-panel figures as (a), (b), (c) rather than "top," "middle," or "bottom" for consistent referencing. 2.2. When citing figures in the text, explicitly note which panel supports each statement (e.g., "Figure 2b shows…"). 2.3. Ensure

> that figure captions are self-contained, describing axes, color coding, and what each panel represents.

Response: It is conventional to label panels with letters, but this is a matter of style, and we feel that descriptors like top, middle, bottom, etc. are unambiguous for the figures in this manuscript. In fact other reviewers noted that the figures are clear. We appreciate the suggestion, but we choose to leave the figures and their captions as is.

> **Reviewer 3:** This manuscript is interesting and useful for operational vaccination activities. It is easy to read, despite a few typos (for example, the word "catagorical"). The figures are legible. The authors should specify the limitations of this study, and the model needs to be validated by analyzing data from several countries.

Response: We thank Reviewer 3 for their attention to detail. We've corrected the typo, and if there are others, we'd be happy to correct those as well.
On the limitations and validation, note that limitations and assumptions are discussed at length in the appendix. Appendices A and B in particular highlight data limitations, related necessary assumptions, and some commentary on the implications for the results. Meanwhile, it would be possible to incorporate data from other countries, but we feel it would distract significantly from the focus in this manuscript. We note however that the modeling approach has been applied to data from other countries, and some published examples (Pakistan and Somalia) are cited in the manuscript (Refs. 6 and 21 respectively).

> **Reviewer 4:** The manuscript was well prepared and technically sound too. The statistical analysis were performed rigorously. The authors too have tried to make the findings available on the manuscript and on GitHub. This manuscript was also written in standard English. However, the conclusion of the research work reflects that Southern Nigeria's 2019 IRI had an effect on measles transmission comparable to its large campaigns. The graphs shown for this work in all forecasting and susceptibility did not present adequate parallel comparison with 2019 and other years in order to support the conclusion. I have also found that this research work was part of a work submitted to an Australian National University

Response: We want to first thank Reviewer 4 for their time and attention.
As far as parallel comparisons between the 2019 IRI and the conventional campaigns go, we feel strongly that the manuscript explores this issue in depth. Specifically, Figure 2 demonstrates the impact over time on susceptibility in Lagos, Figure 4 compares the per-dose impact directly across all 6 interventions, and Figure 5 repeats this comparison across all Southern Nigerian states. Posterior profiles of the type in Figures 4 and 5 are a quantitatively principled method of comparison. The depth and rigor of the approach was noted by Reviewers 1 and 2 as well.
As far as the submission history of this work, the preprint has been on medarXiv for much of this year, but there are no other public versions of the manuscript. The reviewer maybe confusing this work with something else.
